# Dedicated Single-Branch Platforms for Totally Endovascular Zone 2 TEVAR with LSA Revascularization: A Comparison of Castor/Cratos and Gore TAG Thoracic Branch Endoprosthesis

**DOI:** 10.3390/jcm15072659

**Published:** 2026-03-31

**Authors:** Antonio Marzano, Giovanni Gagliardo di Carpinello, Alessia Giordano, Rocco Cangiano, Marta Ascione, Francesca Miceli, Alessia Di Girolamo, Claudia Bittoni, Martina Pacillo, Luca di Marzo, Wassim Mansour

**Affiliations:** Vascular Surgery Unit, Department of General and Specialized Surgery and Anesthesiology, “Sapienza” University of Rome, Viale del Policlinico 155, 00161 Rome, Italy; giovanni.gagliardodicarpinello@uniroma1.it (G.G.d.C.); giordano.2281407@studenti.uniroma1.it (A.G.); rocco.cangiano@uniroma1.it (R.C.); marta.ascione@uniroma1.it (M.A.); francesca.miceli@uniroma1.it (F.M.); alessia.digirolamo@uniroma1.it (A.D.G.); bittoni.1903068@studenti.uniroma1.it (C.B.); pacillo.1785675@studenti.uniroma1.it (M.P.); wassim.mansour@uniroma1.it (W.M.)

**Keywords:** TEVAR, Zone 2, aortic arch, left subclavian artery, Castor, Cratos, Gore TBE, thoracic branch endoprosthesis, aortic dissection, endovascular arch repair

## Abstract

Zone 2 thoracic endovascular aortic repair (TEVAR) frequently requires left subclavian artery (LSA) preservation to maintain vertebrobasilar and upper-extremity perfusion while obtaining a durable proximal seal. Dedicated single-branch endografts were developed to standardize this step and to facilitate a reproducible fully endovascular strategy. Two main device concepts currently shape this field: integrated unibody branch platforms, represented by Castor and the second-generation Cratos, and modular retrograde-branch systems, represented by the Gore TAG Thoracic Branch Endoprosthesis (TBE). The Castor/Cratos evidence base is broader and older, and is mainly centered on type B aortic dissection, with prospective multicenter and real-world data showing favorable branch patency and aortic remodeling. By contrast, TBE evidence is expanding rapidly and is supported by prospective midterm data in arch aneurysms as well as by increasingly large post-commercial series and comparative analyses across zones 0–2. Beyond outcomes, the two platforms differ substantially in branch directionality, potential contribution to proximal fixation, modularity, branch diameter range, proximal landing requirements, access profile, and regulatory/off-the-shelf availability, all of which have direct consequences for anatomical suitability in dissection, aneurysm disease, and trauma. This narrative review synthesizes current evidence and proposes an anatomy-first, pathology-aware framework for selecting between Castor/Cratos and TBE in totally endovascular zone 2 TEVAR with LSA revascularization.

## 1. Introduction

Thoracic endovascular aortic repair (TEVAR) has become the preferred treatment for many lesions of the descending thoracic aorta, including type B thoracic aortic dissection (TBAD), penetrating aortic ulcer (PAU), intramural hematoma (IMH), traumatic aortic injury, and descending thoracic aneurysm [1]. However, the durability of TEVAR remains critically dependent on the quality of the proximal seal [2]. In a substantial proportion of patients, an adequate disease-free proximal landing zone distal to the left subclavian artery (LSA) is not available, and the endograft must therefore be landed in Ishimaru zone 2, inevitably jeopardizing the LSA origin unless dedicated preservation strategies are employed [3,4,5,6,7].

Intentional LSA coverage is not a trivial technical detail. It can alter posterior cerebral circulation, impair collateral pathways to the spinal cord, jeopardize upper-limb perfusion, and create specific risks in patients with prior left internal mammary artery coronary bypass, dominant left vertebral artery, incomplete circle of Willis, or anticipated extensive thoracic coverage [5,6,7,8,9,10]. For that reason, current aortic practice has increasingly moved from routine LSA sacrifice toward selective or systematic preservation, especially in elective cases and in anatomically complex or extensive repairs [7,9,11].

Before the era of dedicated single-branch devices, LSA preservation during zone 2 TEVAR relied on open or hybrid debranching, most commonly carotid–subclavian bypass or transposition, or on purely endovascular workarounds such as chimney/periscope grafting and in situ fenestration. These approaches remain useful, but each carries specific limitations. Hybrid debranching adds cervical dissection, cranial nerve risk, wound morbidity, and an additional operative step, which may be particularly relevant in surgically high-risk or frail patients [12,13]. Chimney techniques introduce the risk of gutters and type Ia endoleaks, whereas fenestration may compromise graft integrity and can be technically demanding, especially in hostile arch anatomy [9,14,15].

Dedicated single-branch platforms were developed to address these shortcomings by incorporating LSA preservation into the primary repair construct itself. However, not all branched devices solve the problem in the same way. Castor (MicroPort Medical, Shanghai, China) and later Cratos (MicroPort Medical, Shanghai, China) use a unibody aortic graft with an integrated antegrade side branch, whereas the Gore TAG Thoracic Branch Endoprosthesis (TBE) (W.L. Gore and Associates, Flagstaff, AZ, USA) uses a modular aortic component with an internal portal and a separate retrograde side-branch component [5,6,7,16,17,18].

This distinction is not merely descriptive and may influence proximal sealing mechanics, branch fixation, freedom from migration, minimum required proximal landing length, branch sizing flexibility, access strategy, and the number of components needed to complete the repair. It also partly explains the different evidence ecosystems in which the devices evolved. Castor was introduced earlier and therefore accumulated a much larger and longer clinical literature, but the overwhelming majority of that literature concerns TBAD and originates from China [5,16,19,20,21,22,23]. By contrast, TBE entered a Western environment dominated by distal arch aneurysm, mixed arch pathology, and regulatory pathways that emphasized prospective feasibility and post-commercial real-world registries [6,7,17,24,25].

Accordingly, the central question is not which device is superior in absolute terms, but rather which platform may be more appropriate for a given anatomy, pathology, and procedural scenario. This review, therefore, focuses on totally endovascular zone 2 TEVAR with LSA preservation and compares Castor/Cratos and TBE through four complementary lenses: anatomical suitability, device design, procedural strategy, and clinical evidence.

## 2. Anatomical Considerations

### 2.1. Zone 2 Sealing: More than a Nominal Landing Length

The success of zone 2 TEVAR depends not only on how many millimeters of “healthy” aorta are available between the Left Common Carotid Artery (LCCA) and the LSA, but also on how that segment behaves in three-dimensional space. The proximal landing segment is short, highly curved, and subject to significant inner–outer curvature mismatch. Arch angulation, calcification, mural thrombus, and the relative apposition of the graft along the outer curvature all influence the likelihood of bird-beak, type Ia endoleak, migration, and adverse aortic remodeling [2,4,9,26,27,28].

These issues become even more pronounced in TBAD. In acute dissection, one is not treating a static aneurysmal lumen but a dynamic geometry with true-lumen compression, tapered distal diameters, branch-vessel relationships driven by the flap, and a proximal landing segment that may be short, fragile, or immediately adjacent to the primary entry tear. This explains why some platforms that perform very well in distal arch aneurysm do not necessarily translate seamlessly into acute dissection, and vice versa [3,4,6,17,26].

This distinction is reflected in the literature. Castor series repeatedly focus on the interplay between proximal seal, remodeling, migration, and branch patency in TBAD [3,4,5,16,19,20,21,22,23,27,29], whereas TBE studies more often evaluate mixed or aneurysmal arch cohorts in which the central question is procedural safety, branch durability, and the capacity to replace hybrid bypass strategies [6,7,17,25].

### 2.2. LCCA–LSA Distance: A Major Discriminator Between Platforms

Perhaps the most important anatomical discriminator between the platforms is the available distance between the distal LCCA and the LSA. For TBE, this distance is critical because the modular portal configuration requires a longer segment of proximal zone 2 to accommodate the portal and covered seal without jeopardizing the LCCA. In the Greek single-center TBE experience, a minimum of 2 cm between the end of the LCCA and the end of the LSA is required for TBE use [30]. Feasibility studies and Instruction For Use (IFU)-based sizing analyses similarly use thresholds around 15–20 mm [28,31].

By contrast, Castor was designed to function in shorter zone 2 anatomies. Its integrated branch can be positioned very close to the proximal edge of the graft, with an offset ranging from 5 to 30 mm. This short-offset design is particularly relevant when the disease extends immediately beneath the LSA or when the LCCA–LSA gap is too short for a modular portal-based solution. Available clinical and feasibility data are broadly consistent with this anatomical interpretation, although such observations should not be read as evidence of platform superiority. Chen et al. specifically treated TBAD with an “insufficient anchoring region” using Castor [3], and Lang et al. reported Castor’s suitability of 82% versus 22% for off-the-shelf TBE in acute TBAD, largely because longer seal requirements excluded many dissection anatomies [26].

This point is central to any realistic selection algorithm. In short-zone anatomies, especially in TBAD, the short proximal configuration of Castor/Cratos may be an important determinant of endovascular feasibility [3,18,26,32]. However, this observation should be interpreted as anatomy-specific rather than as evidence of overall superiority over TBE.

### 2.3. LSA Diameter and Branch Sizing: Why Western Aneurysm Anatomy Matters

The second key anatomical discriminator is the diameter of the target vessel. Castor and Cratos offer branch diameters generally ranging from 6 to 14 mm. This range is entirely appropriate for most TBAD patients and for many Asian series, and it is mirrored by the branch-diameter ranges reported in several clinical studies [16,19,33]. However, it can become limiting in larger, more degenerative proximal subclavian anatomies, particularly in Western aneurysm populations. TBE may be advantageous in this domain because its branch range can accommodate larger target-vessel diameters. TBE provides branch options up to 20 mm, with treated-vessel diameter ranges extending up to 18 mm depending on the portal configuration. This broader branch range is repeatedly emphasized in TBE feasibility studies and technical reviews [28,30,31,34]. As a result, TBE can accommodate larger LSAs without forcing undersizing or reliance on adjunctive relining. This is particularly relevant in degenerative arch aneurysm, in elderly patients, and more broadly in Western populations, where larger supra-aortic vessel diameters are not uncommon.

The practical consequence is that TBE may remain fully endovascular and fully device-based in anatomies in which Castor/Cratos would require an additional covered stent in the side branch to bridge a diameter mismatch. Indeed, in the European Castor feasibility study by Leone et al., large LSA diameter was the single most important cause of non-feasibility, accounting for more than half of all exclusions [32].

### 2.4. Distal Tapering in Acute TBAD: A Core Advantage of Integrated Tapered Platforms

Acute TBAD often creates a marked proximal-to-distal mismatch between the aortic diameter in zone 2 and the distal true lumen. This is one of the main reasons why devices designed for more uniform aneurysmal anatomy may be difficult to size in dissection. Lang et al. showed that, for TBE in acute TBAD, the principal cause of non-feasibility was distal diameter mismatch, usually because the distal aorta was too small for the available TBE configurations [26]. This helps explain why TBE acute TBAD series often require additional distal components and why off-the-shelf suitability in dissection may be limited [17,24,35,36,37].

Castor, by contrast, is available with proximal diameters of 26–44 mm and distal diameters of 20–44 mm, allowing tapered configurations with up to 8 mm of tapering. This may be advantageous in acute TBAD, where pronounced proximal-to-distal mismatch is common. A tapered main body is intrinsically better aligned with proximal sealing in zone 2 and distal treatment in a smaller true lumen. This may partly explain why Castor has accumulated a dissection-heavy literature and why, in comparative anatomical modeling, it has shown broader suitability than TBE in acute e TBAD under strict off-the-shelf assumptions [5,19,20,21,22,23,26,29].

### 2.5. Iliac Access Feasibility and the Concept of “True Feasibility”

Another relevant anatomical issue is whether the patient can physically accommodate the device from an access perspective. This is especially important because thoracic branched devices are bulkier than standard TEVAR systems and because the older, more aneurysmal patients in whom TBE is often used frequently have diseased iliofemoral axes.

Vacirca et al. helpfully separated Anatomical Feasibility (AF) from Iliac Feasibility (IF) and reported a True Feasibility (TF) rate of 85%, down from 92% when access constraints were added. Importantly, access feasibility was significantly lower in women, highlighting that large-profile devices are not evaluated properly if one stops at arch anatomy alone [28]. This point deserves particular attention because the EVAR (Endovascular Aneurysm Repair) literature has consistently shown that women more often present with smaller-caliber access vessels and higher access-related morbidity, including limb ischemia and major bleeding, even in contemporary series; accordingly, sex-aware assessment should ideally be incorporated into the concept of true feasibility in branched arch repair as well [38,39,40].

Most Castor feasibility studies do not formally provide this arch-plus-access analysis. Leone et al. examined morphology in detail but did not define a separate true-feasibility category [32]. Lang et al. focused on arch suitability rather than access suitability [26]. This difference in reporting is relevant because it can make Castor’s suitability appear more favorable on paper when access has not been assessed with the same rigor. At the same time, the profile of the Castor family is often advantageous: the original system uses a 24F sheath, whereas Cratos can drop to 22F in smaller and intermediate sizes [16]. The larger TBE configurations, by contrast, can require up to 26F introducers.

Thus, “true feasibility” remains one of the areas in which the available evidence is still not fully aligned methodologically across platforms [26,28,32].

## 3. Evolution of LSA Preservation in Zone 2 TEVAR

The shift from open or hybrid debranching to fully endovascular LSA preservation has been driven by two parallel needs: reducing the invasiveness of the procedure and standardizing the technical result. Hybrid carotid–subclavian bypass remains durable, but it introduces an extra incision, potential nerve injury, wound complications, and, importantly, a second operative step that partly offsets the minimally invasive advantage of TEVAR. Chimney grafting avoids neck surgery, but gutter-related endoleak remains a structural concern. In situ fenestration is attractive and versatile, but it is operator-dependent, less standardized, and potentially problematic in heavily calcified or highly angulated arches [7,9,14,15].

Dedicated single-branch endografts, therefore, represent the next logical step in the evolution of zone 2 TEVAR. They embed the branch solution within the primary aortic repair and aim to reduce variability. Yet, even within this category, there are two very different philosophies. Castor was the first widely used dedicated single-branch zone 2 thoracic device and was initially developed to preserve the LSA in TBAD. Its strong association with dissection is therefore historical as well as anatomical [5,19,20,23]. TBE emerged later in a Western regulatory and commercial context, with a broader focus on arch pathology and with immediate relevance not only to zone 2 but also to zones 0 and 1 through a modular branch concept and proximal extension options [6,7,17,34].

This divergence in origins matters because it has shaped the evidence base. The Castor literature is larger but less geographically diverse; the TBE literature is newer but more Western and more heterogeneous in pathology. Any meaningful comparison between them must therefore distinguish between device performance and the clinical ecosystem in which the device was adopted [5,6,7,17,25].

## 4. Device Design Comparison

### 4.1. Unibody Branch Versus Modular Retrograde Branch

The most fundamental distinction is architectural. Castor and Cratos are unibody devices in which the aortic component and the side branch are part of a single integrated structure. TBE is a modular system consisting of an aortic component with an internal portal and a separate side-branch component [6,34]. This design distinction influences almost every downstream aspect of the procedure.

In Castor/Cratos, the side branch is deployed together with the main graft, and the branch is oriented in an antegrade, more physiologic direction. In TBE, the branch is inserted retrogradely through a portal and therefore functions as an additional modular component [6,17,25,34]. This is not simply a matter of elegance. Castor/Cratos behaves as a single integrated construct, whereas TBE inherently introduces at least one component junction even before any distal aortic extensions are added. In addition, it requires deployment of a separate bridging branch into the LSA, which increases procedural complexity and may expose the repair to component-related failure modes such as malalignment, junctional endoleak, or loss of apposition. Similar concerns are well known from other multicomponent endovascular systems [41], including fenestrated and branched endovascular aneurysm repair [42,43,44,45].

### 4.2. Active Fixation and Sealing Contribution of the Integrated Branch

One conceptual distinction of Castor/Cratos is that the integrated side branch may contribute not only to LSA perfusion but also to proximal construct stabilization. Because the side branch is part of the main graft, it may provide an anchoring vector into the target vessel. This may be particularly relevant in dissection or aneurysm, where the proximal seal is short, the aortic wall is vulnerable, and migration or displacement may develop even when the immediate angiographic result looks acceptable. This hypothesis is supported indirectly by clinical evidence. In the acute TBAD comparison by Liu et al., Castor had 0% stent displacement versus 20% with standard TEVAR and markedly lower reintervention rates at 2 years [27]. Although this was not a direct Castor-versus-TBE study, it supports the idea that an integrated branch can stabilize the proximal zone 2 repair. Likewise, Chen et al. reported favorable early results in very short anchoring zones, again supporting the notion that Castor’s side branch is functionally relevant to proximal fixation [3].

By contrast, in TBE, the side branch primarily serves flow preservation, while proximal sealing depends mainly on the covered aortic component and its relationship with the native arch anatomy. The branch is retrograde and modular, and the proximal seal is determined by the covered aortic component and its relation to the native arch. The side branch preserves flow, but it does not meaningfully reduce the proximal landing requirement, which remains a core anatomical constraint in the TBE system [28,31].

Whether these architectural differences translate into clinically meaningful differences in fixation or migration risk remains to be demonstrated in direct comparative studies.

### 4.3. Modularity: Versatility on One Side, More Overlap Zones on the Other

The main advantage of TBE modularity is versatility. The system can be configured across zones 0, 1, and 2, and the same fundamental aortic component/side-branch logic can be used in different arch scenarios [6,7,17,25,34]. This is a major practical strength, particularly in distal arch aneurysm, prior ascending replacement, or more proximal arch disease. The downside is equally clear: modularity means more components, more interfaces, and often more adjunctive extensions. The real-world TBE literature repeatedly shows this. Distal extensions were common in the Dutch single-center European experience [36], in the large Cleveland Clinic post-commercial series [17], in Pang’s zone 0–2 cohort [25], and in the urgent Padua case series [37]. Each additional overlap zone may represent a site of malapposition, kinking, or device-related failure, as recognized in other branched aortic platforms [41,42].

The same principle applies to the bridging side-branch component. TBE branch durability is generally excellent [6,7,25], but the system still requires an additional modular connection and often additional distal thoracic components, whereas Castor/Cratos aims to reduce the number of moving parts at the arch level [6,7,17,25,34].

### 4.4. Proximal Offset and Short-Neck Advantage

Another decisive design feature is the proximal branch offset. In the Castor/Cratos system, the distance from the proximal edge of the main body to the branch can range from 5 mm to 30 mm. This allows very short proximal configurations and makes the device usable in anatomies with minimal space between the LCCA and the LSA.

TBE does not have this degree of short-offset flexibility. Its modular portal design requires a more generous proximal seal and has translated in practice into threshold distances of roughly 15–20 mm in feasibility work, or around 2 cm in single-center clinical interpretation [28,30,31]. This difference represents a potentially relevant anatomical distinction, particularly in short LCCA–LSA configurations and dissection-centered anatomies [26].

### 4.5. Branch Diameter Range: A Real Advantage for TBE in Larger LSAs

If Castor/Cratos may be better aligned with very short zone 2 anatomy, TBE may be better suited to large-LSA anatomy. Branch diameters up to 20 mm allow TBE to accommodate larger target vessels that would exceed the intended Castor side-branch range [28,30,31]. In practical terms, this is often more relevant in degenerative aneurysm disease than in acute TBAD. Many dissection patients have target-vessel diameters well within the Castor range, which partly explains the strong Castor experience in that setting. However, once the LSA diameter exceeds roughly 13–14 mm, the integrated-branch platform may require adjunctive relining or may become anatomically unsuitable [32].

Thus, branch diameter is not just a sizing detail, but a pathology-specific selection factor: Castor is usually adequate for dissection, whereas TBE is often more forgiving in aneurysmal or large-caliber supra-aortic anatomy [28,32].

### 4.6. Delivery Profile and Access

The delivery profile comparison also deserves a nuanced reading. The classic Castor system uses 24F across its range, whereas Cratos reduces this to 22F up to 34 mm in the multicenter second-generation study [16]. TBE, depending on size, may require 20F to 26F introducers. This means that smaller TBE configurations can be comparable or even advantageous, but larger TBE configurations become less favorable from an iliac-access standpoint, especially in aneurysm patients and women [28].

Upper-extremity access goes in the opposite direction. Castor/Cratos generally requires 7–8F branch access [5,33], whereas TBE commonly uses more limited brachial or axillary access, often around 4–5F in practice [30,37]. This distinction is not negligible, because larger upper-extremity sheaths may more frequently necessitate surgical exposure to mitigate access-related complications [46]. Therefore upper-extremity access strategy should be considered an integral part of procedural planning rather than a secondary technical detail. Thus, TBE may be easier from the arm and harder from the groin, whereas Castor/Cratos may be more favorable from the groin, particularly in the Cratos generation, but more demanding at the supra-aortic access site.

### 4.7. Maneuverability and the Cratos Response to First-Generation Limitations

One legitimate criticism of first-generation integrated devices is maneuverability. Castor requires careful rotational alignment during advancement, and the learning curve can be non-trivial. The Scandinavian multicenter study is particularly informative here: the authors explicitly describe wire wrapping as a potential issue during advancement, emphasize the need for familiarity with the system, and report one technical failure caused by displacement during off-IFU zone 1 plus laser-fenestration manipulation. They also document one episode of premature release of the branch cap, which was corrected by removing and remounting the device before successful implantation [11]. These observations confirm that first-generation Castor is technically elegant but operator-sensitive.

Cratos appears to address many of these limitations. Li et al. reported that the second-generation system incorporates design refinements aimed at improving orientation control and proximal conformability (the delivery system features an additional dedicated trigger that allows control of proximal apposition and helps mitigate bird-beak formation), with encouraging early safety and 12-month branch patency [16]. The first Western Cratos case also suggested improved rotational control and a more favorable delivery profile [18]. A recent technical note further details these modifications, including an adjustable proximal segment, active bird-beak control, and reinforcement of the branch-main body junction [47]; nonetheless, these features should still be regarded as promising technical refinements rather than clinically established advantages until supported by larger series and longer follow-up.

## 5. Procedural Strategies

### 5.1. Preoperative Planning and Device Selection

**Figure 1:**
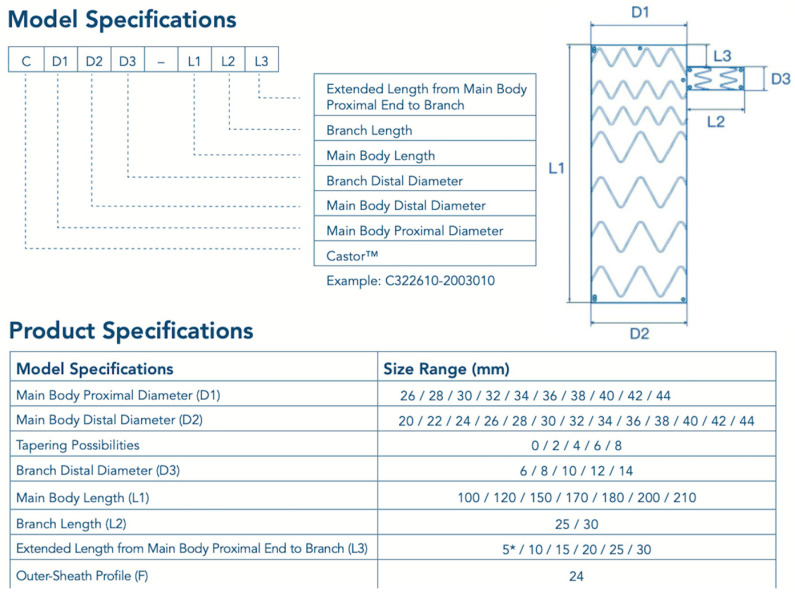
Available Castor/Cratos configurations (* The device’s effective length is 7 mm).

**Figure 2:**
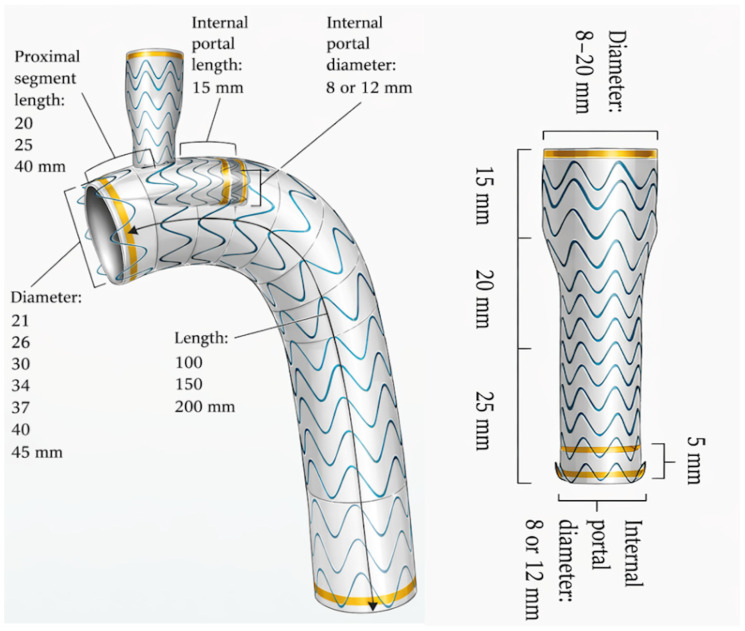
Available Gore TBE configurations.

For both device families, preoperative Computed Tomography Angiography (CTA) remains the essential planning modality. At a minimum, the following variables should be carefully assessed in order to guide optimal device selection for Zone 2 implantation [26,28,31,32]:

1. Proximal landing-zone diameter and length on the outer curvature, inner curvature, and centerline;
2. LCCA–LSA distance, specifically the distance from the distal edge of the LCCA to the proximal edge of the LSA (critical for Castor/Cratos), the distance from the distal edge of the LCCA to the distal edge of the LSA, and the distance from the center of the LCCA ostium to the distal edge of the LSA (both key measurements for TBE); in all cases, these measurements should be obtained along the outer curvature;
3. LSA diameter and the length of the prevertebral segment;
4. Distal thoracic diameter;
5. Iliac-femoral access diameters, calcification, and tortuosity;
6. The different device configurations are illustrated in Figure 1 for Castor and Cratos, and in Figure 2 for TBE.

The diameters and lengths covered by the available configurations of the different devices are summarized in Table 1.

### 5.2. Device Implantation Procedures

Based on the largest available clinical series [6,16,18,21,33,34], the most practical procedural setup is generally, under general anesthesia, a three-access strategy, consisting of two femoral accesses (one main working access and one ancillary access) and one left upper-extremity access. For Castor/Cratos, because the upper-extremity branch access typically ranges around 7–8F, several authors have favored a small surgical brachial cutdown in order to minimize access-related complications. By contrast, Gore TBE usually requires a smaller upper-extremity working access, commonly around 4–5F in routine practice, and can therefore often be managed through simple percutaneous radial or brachial access. As for ballooning, proximal molding of the main graft is generally left to the operator’s discretion, as is distal ballooning, unless the distal sealing zone lies within another implanted device or across a graft overlap. With regard to the subclavian branch, routine ballooning is not mandatory for Castor/Cratos, although it is advisable in the presence of branch kinking or suboptimal expansion. By contrast, for TBE, the Instructions for Use recommend systematic ballooning of the side-branch component at three levels: at the overlap zone within the retrograde internal portal, along the mid-portion of the branch component, and distally within the LSA. Finally, deployment of the main graft is strongly recommended under controlled hypotension and bradycardia.

#### 5.2.1. Castor Implantation Technique

A through-and-through wire is usually established between the left upper-extremity access, most commonly through a 7F brachial sheath, and one femoral access, typically the right, using a snaring maneuver. After the guidewire has been externalized, the branch catheter is advanced from the upper-extremity access and connected to the pre-flushed side branch of the device. The main graft, mounted on a 24F delivery system, is then introduced from the femoral access and advanced into the arch under fluoroscopic guidance. Particular attention is paid to maintaining coaxial alignment and to preventing wire twisting during advancement. The radiopaque markers are used to confirm correct rotational orientation, with the branch directed cranially toward the LSA origin. If malrotation or wire entrapment is recognized, the device can be withdrawn into the sheath and carefully reoriented before re-advancement. Final deployment is usually performed under controlled pharmacological hypotension and bradycardia in order to improve positional accuracy. Release of the branch into the LSA is then completed under tension on the upper-extremity wire, allowing precise engagement and stabilization of the branch component. The overall procedural workflow is summarized in Video S1.

#### 5.2.2. Cratos Implantation Technique

A through-and-through brachiofemoral wire is established using standard snaring techniques. After placement of a stiff guidewire into the ascending aorta, the delivery catheter introduced from the brachial access is retrieved through the femoral sheath and docked into the pre-flushed Cratos branch. The main graft, pre-mounted on a 24F or, in selected configurations, a 22F delivery system, is then advanced from the femoral access. Radiopaque markers arealigned with the LSA origin under fluoroscopic guidance. The improved rotational control of the Cratos introducer, positioned in the proximal descending thoracic aorta, allows fine orientation adjustments even in the presence of marked tortuosity. In cases of malrotation, the device can be partially resheathed within the introducer and safely repositioned before redeployment. After release of the main graft through the dedicated deployment knob, the delivery system features an additional dedicated trigger that allows control of proximal apposition and helps mitigate bird-beak formation. Controlled tension on the brachial wire then enables accurate release of the branch into the LSA. The overall procedural workflow is summarized in Video S2.

#### 5.2.3. TBE Implantation Technique

From the left brachial access, a guidewire is advanced and then retrieved from the right femoral access, thereby creating a left brachiofemoral through-and-through wire. Over a stiff aortic guidewire and with the brachiofemoral rail in place, the main device is advanced from the primary femoral access (typically 20–26 Fr, depending on the selected configuration) into the intended position and deployed after angiographic confirmation. Release is achieved using the dedicated deployment handle in a single-step maneuver. During this phase, maximal control of the delivery system is essential to prevent inadvertent forward or backward movement of the graft. The dedicated branch component for the left subclavian artery is subsequently advanced over the brachiofemoral through-and-through wire, deployed, and balloon-molded. The overall procedural workflow is summarized in Video S3.

### 5.3. Deployment Pitfalls

For Castor/Cratos, rotational alignment and avoidance of wire wrapping are central.

For TBE, the main procedural issue is not so much the rotation of a unibody graft but the orchestration of a modular system: proper portal alignment, safe branch advancement, sufficient overlap, and careful planning of any distal extensions. This complexity is manageable, but it means that “totally endovascular” does not necessarily mean “single-device” or “simple.”

### 5.4. Completion Imaging and Surveillance

At completion of angiography, the operator should document:

- Absence of type Ia/Ib/Ic/III endoleak;
- Branch patency;
- Branch geometry and absence of kinking;
- Absence of displacement or malapposition;
- Integrity of any modular overlaps or distal extensions [3,5,6,11,17,25].

**Figure 3:**
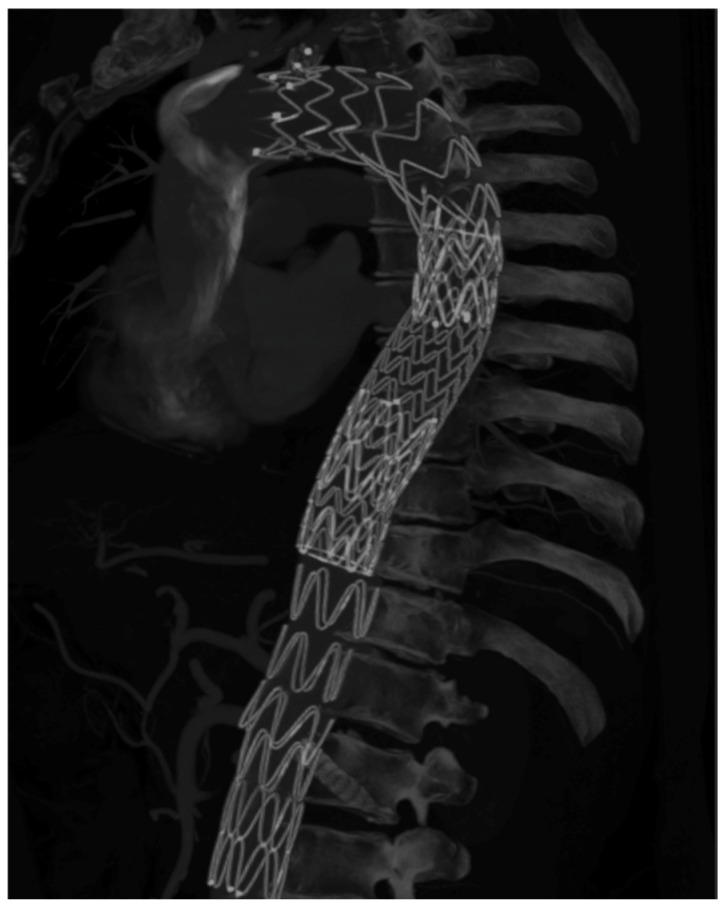
Postoperative 3D CTA reconstruction following Castor implantation combined with the STABILISE technique.

**Figure 4:**
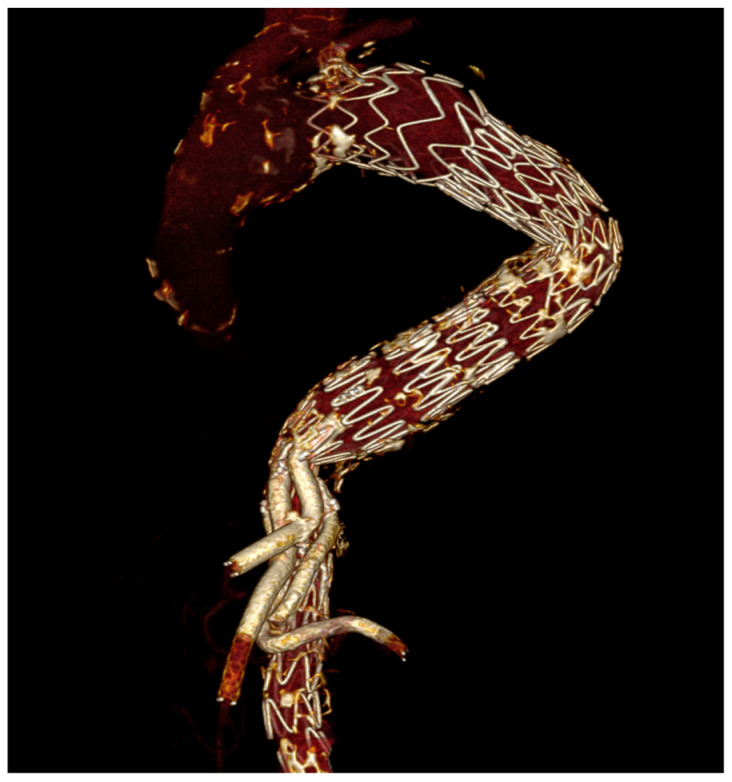
Postoperative 3D CT reconstruction following Cratos implantation combined with branched endovascular aortic repair (BEVAR).

**Figure 5:**
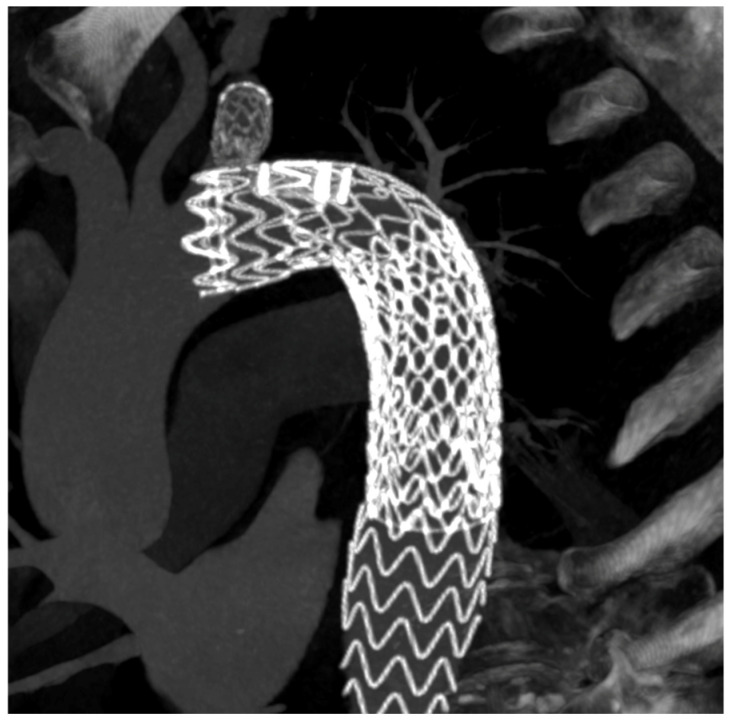
Postoperative 3D CT reconstruction after TBE implantation combined with a distal straight thoracic stent-graft module.

Follow-up CTA is particularly important in dissection because the immediate goal is not merely technical success but durable remodeling. Several Castor studies provide serial remodeling data, including false-lumen thrombosis and true-lumen expansion [5,19,20,22,29,33]. TBE follow-up is more often reported in terms of branch durability, survival, and reintervention, especially in aneurysm cohorts [6,7,17,25]. Figure 3, Figure 4 and Figure 5 show postoperative 3D CT reconstructions of a Castor, a Cratos, and a TBE, respectively.

Specifically, Figure 3 exemplifies the integrated unibody Castor configuration in a dissection setting combined with the STABILISE (Stent-Assisted Balloon-Induced Intimal Disruption and Relamination in Aortic Dissection Repair) technique; Figure 4 illustrates the second-generation Cratos configuration within a complex branched endovascular repair; and Figure 5 shows the modular TBE architecture with a distal straight thoracic extension component.

## 6. Clinical Evidence

### 6.1. Literature Identification Strategy

This review was designed as a structured narrative review rather than a formal PRISMA systematic review. This approach was chosen because the available literature on dedicated single-branch platforms for totally endovascular zone 2 TEVAR is highly heterogeneous in study design, pathology, objectives, and reported endpoints, and because the purpose of the present work was not to generate pooled comparative estimates, but to provide a clinically oriented synthesis of anatomy, device design, procedural strategy, and pathology-specific applicability.

A literature search was performed in PubMed and Scopus up to 25 February 2026 using combinations of the following keywords: “*thoracic branch endoprosthesis*”, “*Gore TBE*”, “*single-branched TEVAR*”, “*Castor stent graft*”, “*Cratos stent graft*”, “*zone 2 TEVAR*”, “*left subclavian artery preservation*”, “*LSA revascularization*”, “*aortic dissection involving the left subclavian artery*”, “*distal arch aneurysm*”, and “*blunt thoracic aortic injury*”. In addition, the reference lists of major clinical series, anatomical feasibility studies, and relevant review articles were screened manually to identify further eligible publications.

Eligible studies were English-language reports specifically focused on dedicated single-branch platforms used for totally endovascular arch repair involving zone 2 with LSA preservation. These included clinical implantation series, comparative observational cohorts, anatomical feasibility studies, meta-analyses, technical reports with direct clinical relevance, and selected high-relevance case reports when they provided unique information on new-generation devices or Western experience. Non-English articles, duplicate reports, non-clinical publications, and studies not specifically addressing dedicated zone 2 single-branch platforms with LSA preservation were excluded.

Study selection and data extraction were performed by the authors through full-text review, with particular attention to pathology treated, anatomical applicability, technical success, branch patency, reintervention, remodeling data when available, and device-specific procedural considerations. To avoid conflating anatomical suitability with clinical performance, anatomical feasibility studies were analyzed separately from implantation-outcome studies.

No formal risk-of-bias or study-quality scoring tool was applied. This was considered more appropriate for a structured narrative review because the final evidence base included markedly heterogeneous study types, ranging from feasibility analyses and retrospective case series to comparative cohorts, meta-analyses, and isolated case reports. This methodological choice represents an inherent limitation of the review and is acknowledged as such in the discussion of the evidence base.

The final dataset included 33 studies [3,4,5,6,7,9,11,16,17,18,19,20,21,22,23,24,25,26,27,28,29,30,31,32,33,34,35,36,37,47,48,49,50]. The implantation-outcome studies are summarized in Table 2. Feasibility studies were analyzed separately from implantation-outcome studies to avoid conflating anatomical suitability with procedural or clinical durability [26,28,31,32] and are summarized in Table 3.

### 6.2. Two Evidence Ecosystems Rather than One Head-to-Head Literature

The most important interpretive point is that Castor/Cratos and TBE do not yet share a balanced, directly comparable evidence environment.

The Castor/Cratos literature is clearly broader and older. It includes a prospective multicenter trial with long-term follow-up [5], multiple large retrospective or real-world multicenter series [16,19,20,21], midterm and long-term remodeling data [22,29], and a systematic review/meta-analysis focused on TBAD [23]. However, this evidence is overwhelmingly dissection-driven and largely Chinese. Western experience exists, but it remains far more limited and includes mainly small elective cohorts, case reports, and a few multicenter European studies with relatively short follow-up [11,18,49,50].

The TBE literature is different. It is younger but more geographically Western. It is anchored by the prospective feasibility trial in aortic arch aneurysm [6] and has rapidly expanded through post-commercial single-center, multicenter, and comparative real-world studies [7,17,24,35,37,48]. It also includes a substantial amount of acute-pathology and trauma evidence [24,31,35,37,48]. In other words, TBE was not first adopted primarily in dissection but rather in a broader arch-repair landscape.

This matters because many apparent differences between Castor/Cratos and TBE likely reflect differences in pathology, anatomy, inclusion criteria, and study design rather than intrinsic device superiority. Practice setting may still contribute indirectly, insofar as device availability, regulatory pathways, referral patterns, and population morphology are not identical across regions [51,52,53,54]; however, geography itself should not be interpreted as an independent determinant of device performance.

A platform that looks excellent in acute TBAD may not have been exposed equally to large aneurysmal arches; a platform with elegant aneurysm data may not yet have been stress-tested across the full acute-dissection anatomical spectrum [5,6,7,23,26].

### 6.3. Castor/Cratos: Strengths and Limits of the Current Evidence

The strongest component of the Castor literature is unquestionably TBAD. The multicenter prospective trial by Jing et al. remains a landmark study, with 73 patients, high technical success, no perioperative stroke, excellent 6-year survival, and durable branch patency [5]. This is complemented by large retrospective series showing technical success above 95%, low neurological event rates, and meaningful midterm follow-up [19,20,21]. The meta-analysis by Yao et al. further consolidates this picture, reporting pooled technical success of 97.5%, perioperative stroke of 0%, and 30-day mortality below 1%, although all pooled evidence remained observational and of low to very low certainty [23].

Castor has also shown consistent remodeling effects. Tian et al. reported long-term patency and favorable false-lumen thrombosis out to nearly six years [22]. Yuan et al. showed clear true-lumen expansion and false-lumen reduction, while also drawing attention to the fact that distal abdominal remodeling may be less predictable than thoracic remodeling [29]. These studies collectively reinforce the idea that Castor is not merely a branch-patency device but a dissection-specific remodeling platform [5,19,20,22,23,29].

However, the limitations are equally important. Most data come from China, where practice patterns, patient anatomy, and regulatory access are not identical to Western settings [5,16,19,20,21,23]. Western Castor evidence remains fragmentary. The Italian single-center experience by Rizza et al. included only ten patients and was elective and custom-made [50]. The Polish multicenter study by Żołnierczuk et al. showed encouraging feasibility but also a 4.8% Retrograde Type A Dissection (RTAD) rate and type I endoleak/bird-beak rates of nearly 10% [49]. The Scandinavian multicenter study fills an important gap by providing the largest contemporary European Castor series: 23 patients, 96% technical success, 95% side-branch patency, no 30-day major adverse events, but also 23% bird-beak and two reinterventions for distal stent graft–induced new entry tears, underlining that Western experience is improving but still short-term and not free of device- or anatomy-related failure modes [11].

Cratos, finally, remains promising but still immature. The second-generation multicenter study suggests improved profile, maneuverability, and conformability [16], the first Western implantation supports this impression [18], and a recent technical note highlights further design refinements for proximal sealing and active bird-beak control [47]; however, long-term Cratos-specific evidence is still lacking.

### 6.4. TBE: Strengths and Limits of the Current Evidence

TBE’s most solid foundation remains the prospective multicenter feasibility trial in distal arch aneurysm, which demonstrated 100% technical success, zero 30-day mortality, 93% branch patency at 3 years, and excellent freedom from rupture, migration, and reintervention [6]. This is a high-quality benchmark study and remains one of the most important arguments in favor of TBE in aneurysmal distal arch disease.

Post-commercial experience has broadened the picture. Pang et al. described early outcomes across zones 0–2, with 100% technical success, acceptable 30-day mortality, and preserved branch patency, but also frequent need for distal extensions and proximal cuffs [25]. Lou et al. extended this by showing that post-commercial TBE can be applied broadly in real-world patients, including mixed aneurysm and dissection anatomy, albeit with a notable 16.4% endoleak rate and frequent adjunctive procedures [17]. Most importantly from a comparative-practice standpoint, Satam et al. demonstrated in a zone 2 comparison that TBE was associated with lower Myocardial Infarction (MI) and Acute Kidney Injury (AKI), shorter procedures and hospitalization, greater sac regression, and better freedom from reintervention than TEVAR combined with carotid–subclavian bypass [7].

TBE’s acute-pathology evidence is also growing. DiLosa and colleagues reported multicenter and single-center acute-pathology experiences [24,35], while Spertino et al. demonstrated feasibility in urgent acute aortic syndromes, albeit with universal use of distal extensions [37]. Trauma-specific evidence is another TBE strength: the device has both comparative clinical data against standard TEVAR with LSA coverage and dedicated anatomical feasibility modeling in Blunt Thoracic Aortic Injury (BTAI) [31,48].

The limitations of TBE are different from those of Castor. The device is not hampered by a lack of Western data, but much of its real-world literature still has relatively short follow-up [17,24,25,35,36,37]. Its data are also more heterogeneous in pathology and zone, making it harder to isolate “pure zone 2” performance from broader arch-repair experience [7,17,25]. Finally, the acute TBAD literature remains less mature than the Castor literature, and anatomical feasibility in dissection is lower when strict off-the-shelf criteria are applied [26].

The main advantages and limitations of the different devices are summarized in Table 4.

## 7. Patient Selection

### 7.1. Clinical Scenarios in Which Castor/Cratos May Be Particularly Suitable

First, Castor/Cratos may be particularly suitable in acute or subacute TBAD with inadequate proximal landing or minimal LCCA–LSA distance. This is the setting in which its evidence is strongest and its design is most logically aligned with the anatomy [3,4,5,16,19,20,21,22,26,27,29].

Second, Castor/Cratos is attractive when the operator wants a more integrated construct with fewer modular arch junctions. The lower displacement and reintervention rates seen versus standard TEVAR in acute TBAD support this preference [27].

Third, Cratos may be especially useful in patients with borderline iliac access because of its smaller delivery profile up to 34 mm [16,18].

### 7.2. Clinical Scenarios in Which TBE May Be Particularly Suitable

First, TBE may be particularly suitable in distal arch aneurysms and in mixed elective arch pathology where larger branch diameters and a formally broader arch role are important [6,7,17,25].

Second, TBE is preferred when the target vessel is large, particularly when the LSA diameter approaches or exceeds the comfortable range of Castor [28,30,31,32].

Third, TBE is highly attractive when the goal is to avoid carotid–subclavian bypass in a patient who is anatomically suitable for a fully endovascular zone 2 solution, because the strongest comparative real-world data against hybrid bypass currently support TBE [7].

Fourth, TBE has a more relevant trauma-specific evidence base than Castor [31,48].

### 7.3. Practical Nuance: Regulatory Off-the-Shelf Status Versus Real-World Logistics

TBE is unequivocally an off-the-shelf modular platform, with formal approval from the Food and Drug Administration (FDA) and European Conformity (CE) marking under the Medical Device Regulation (MDR), and its use in the contemporary arch literature already spans zones 0, 1, and 2 [6,7,17,34].

Castor/Cratos occupies a more nuanced position. In daily practice, particularly in markets with high experience and established distributor logistics, operators may experience the platform as functionally close to off-the-shelf because many configurations exist and can be sourced relatively quickly. However, the currently available European Castor documentation still defines the device as custom-made and available on a named-patient basis only, whereas in China—where it was approved in 2017 by the China Food and Drug Administration (CFDA), now the National Medical Products Administration (NMPA)—it has effectively functioned as an off-the-shelf solution in routine practice. The Scandinavian multicenter experience likewise described Castor as a custom-made device, with delivery times generally around 2 weeks and longer for less common configurations. This regulatory and logistical difference is not trivial, as it may restrict acute applicability outside highly specialized centers and remains a relevant practical distinction from TBE [11].

## 8. Limitations of the Evidence

Several limitations must be acknowledged.

First, the literature remains strongly pathology-imbalanced. Castor/Cratos is primarily a TBAD literature; TBE is primarily a Western mixed-arch and aneurysm literature [5,6,7,17,23,25].

Second, the evidence base is unevenly distributed across practice settings. Most mature Castor data come from Chinese centers, whereas most TBE data come from North American and European centers. We refer to this difference only as a contextual proxy for variation in regulatory availability, case mix, referral pathways, and possibly population morphology, not as an independent explanation of device performance.

Third, follow-up remains asymmetric. Castor has long-term dissection follow-up [5,22], whereas many TBE studies remain shorter-term outside the prospective aneurysm trial [7,17,24,35,36,37].

Fourth, comparative data are observational, not randomized [7,9,48]. Thus, differences in urgency, pathology, anatomy, and institutional experience likely influence the results.

Fifth, there is still only limited Cratos-specific evidence [16,18,47]. Therefore, some of the advantages attributed to Cratos, particularly regarding maneuverability and profile, remain early and should not yet be overinterpreted.

Additionally, this work was conceived as a structured narrative review rather than a formal systematic review; therefore, no formal risk-of-bias assessment was performed, and the synthesis should be interpreted as qualitative and clinically oriented rather than as a graded comparative evidence assessment.

## 9. Future Perspectives

The next phase of this field should move in four directions.

- The first is standardized anatomical reporting. Every study on branched zone 2 TEVAR should report, at minimum, LCCA–LSA distance, LSA diameter, LSA–vertebral artery distance, proximal and distal aortic diameters, tapering, access-vessel diameters, and whether true feasibility includes iliac assessment [26,28,31,32].
- The second is a pathology-stratified comparison. Castor/Cratos and TBE should not be compared globally but within clearly separated groups: acute TBAD, chronic dissecting aneurysm, distal arch aneurysm, PAU/IMH, and trauma.
- The third is longer-term Western data, especially for Castor/Cratos and especially for Cratos. The Scandinavian, Polish, and Italian experiences are encouraging but still too short and too small to balance the Western TBE literature convincingly [11,49,50]. Conversely, TBE needs more mature acute-dissection-specific data before its role in acute dissection can be defined with the same degree of confidence currently supported for Castor-based series.
- Finally, future refinements of the TBE platform may also involve improved delivery-system control. In particular, a potential strategy to reduce forward or backward device jumping during deployment and to improve control of proximal bird-beak formation could be the incorporation of features conceptually similar to those of the Gore TAG Conformable Thoracic Stent Graft (W.L. Gore and Associates, Flagstaff, AZ, USA), which was developed to enhance deployment precision and proximal conformability.

## 10. Conclusions

Dedicated single-branch endografts have transformed zone 2 TEVAR from a frequently hybrid procedure into a genuinely totally endovascular strategy in appropriately selected patients. However, Castor/Cratos and TBE are not interchangeable solutions to the same problem.

Castor/Cratos currently has the largest and most mature clinical literature, with the clearest strength in type B aortic dissection, where short proximal landing zones, distal tapering, and the need for durable remodeling make the integrated branch concept especially attractive. Yet this literature is predominantly Chinese, and Western data remain limited to small, elective, or early multicenter experiences.

TBE, in contrast, is a modular, truly off-the-shelf Western platform with formal multi-zone arch applicability and excellent branch-diameter flexibility. Its strongest data come from distal arch aneurysm and mixed real-world arch pathology, where it has shown durable midterm branch performance and, in zone 2, a clear advantage over hybrid carotid–subclavian bypass in contemporary comparative practice.

Accordingly, the most rational contemporary approach remains anatomy-first and pathology-aware. In short zone 2 anatomies and dissection-dominant morphologies, Castor/Cratos may align well with the underlying anatomical constraints. In large-caliber LSA anatomy, aneurysmal disease, or settings where a widely available off-the-shelf modular solution is needed, TBE may be particularly suitable.

The real question is therefore not whether Castor/Cratos or TBE is universally superior, but which one better matches the patient in front of the operator.

## Figures and Tables

**Table 1:** The table summarizes the diameters and lengths covered by the different device configurations, together with the delivery-system profiles required for implantation.

Parameter	Castor/Cratos	TBE
Intended proximal aortic treatment range	22–41 mm	16–42 mm
Intended distal aortic treatment range	16–41 mm	16–42 mm
Tapering within branched component	Up to 8 mm	Not available
Branched component length	100–210 mm	100/150/200 mm
Proximal branch offset/short-zone capability	5–30 mm	Longer portal-based geometry required
LSA diameter covered	5–13 mm (branch 6–14 mm)	5–18 mm (side branch 8–20 mm)
Prevertebral/branch landing length	25–30 mm (European Market)	25–30 mm
Internal portal options	-	8 or 12 mm (14F)
LCCA–LSA spacing required	≥5 mm	≥15 mm short distance ≥20 mm long distance
LSA–LVA distance required	>25 mm	>25 mm
Aortic delivery profile	24F Castor; 22F Cratos up to 34 mm	20–26F
Upper-extremity access	7–8F	4–5F

**Table 2:** Implantation-outcome studies included in the review and their key findings.

Ref.	Device	Study Type	N	Main Pathology	Main Findings
[3]	Castor	Single-center retrospective	26	TBAD with insufficient anchoring region	Technical success: 100%. Stroke: 0%. Paraplegia: 0%. LSA stenosis/occlusion: 0%. Retrograde progression/reverse avulsion: 0%. Type I endoleak: 2/26 (7.7%), both resolved during follow-up. Mean follow-up: 24 ± 6.4 months. Favorable thoracic aortic remodeling during follow-up
[4]	Castor	Single-center retrospective	41	TBAD without proximal landing zone	Technical success: 97.6%. Perioperative mortality: 0%. Stroke: 0%. Paraplegia: 2.4%. Median follow-up: 6 months. 30-day aortic-related adverse events: 4.9%. Bird-beak at 30 days: 14.6%. 6-month aortic-related adverse events: 26.8%. Bird-beak at 6 months: 29.3%. Type Ia endoleak at 6 months: 9.8%. Stent migration at 6 months: 14.6%. Outcomes were worse when LCCA-LSA distance was ≤10 mm: adverse events 44.4% vs. 13.0% and bird-beak 55.6% vs. 8.7%
[48]	TBE vs. standard TEVAR	Single-center retrospective	65	BTAI ^1^	Technical success: 100% in both groups Complications: 0% vs. 25% Aortic-related mortality: 0% TBE side branch patency: 100% Mean follow-up: 155 vs. 364 days Procedure time: 88.5 vs. 102 vs. 267.2 min (*p* < 0.001)
[24]	TBE	Multicenter retrospective	107	Acute aortic pathology	Technical success: 99%. 30-day mortality: 2%. Major adverse events: 18%. Stroke: 6%. Paraplegia: 6%. RTDA ^2^: 5%. Mean follow-up: 55 ± 171 days. Follow-up imaging available in 94%. Type IA/III endoleak: 7%. Retrograde branch occlusion: 1%. Reintervention: 10%. Cumulative aortic-related mortality: 3%.
[35]	TBE	Single-center retrospective	20	Acute aortic pathology	Technical success: 100%. Exclusion of pathology: 100%. LSA patency: 100%. Major complications within 30 days: 0%
[36]	TBE	Single-center retrospective	20	Mixed	Median age: 72 years; male sex: 50%. Median follow-up: 6 months (1–12). In-hospital mortality: 0%. 30-day mortality: 0%. Branch patency at 6-week CTA: 100%. Type I/III endoleak at 6-week CTA: 0%. Stroke: 0%. Paraplegia/paraparesis: 0%. Secondary intervention for branch-related type III endoleak: 1/20 (5%), successfully treated with adjunctive stenting. Type II endoleak: 2/20 (10%), without aneurysm growth. Post-discharge sudden deaths of unknown cause: 2/20 (10%) at 7 and 8 weeks; CTA at 6 weeks showed no aortic/TBE abnormality in either patient.
[5]	Castor	Multicenter prospective	73	TBAD	Technical success: 97% (71/73). Intraoperative endoleak: 5% (4/73). In-hospital mortality: 1.4% (1/73). Major complications: 0%. Median follow-up: 61 months (48–72). 1-year mortality: 5% (4/73). 6-year mortality: 7% (5/73). Branch-section patency at follow-up: 93% (63/68). Reintervention for new entry tears: 2 cases. Residual intraoperative endoleaks resolved during follow-up
[30]	TBE	Single-center retrospective	12	Mixed	Technical success: 100%. LSA side-branch patency: 100% perioperatively. Final angiographic type I/III endoleak: 0%. 1-month follow-up available in 10/12 patients. Type II endoleak at 1 month: 1/10 (10%), without sac enlargement. 6-month CTA available in 7 patients. Type I/III endoleak at 6 months: 0%. Stent migration at 6 months: 0%. Stent fracture at 6 months: 0%.
[19]	Castor	Single-center retrospective	150	TBAD	Technical success: 97.3% (146/150). Intraoperative RTAD ^1^: 0.7% (1/150). Type I endoleak: 1.3% (2/150). Stroke: 0.7% (1/150). In-hospital aorta-related mortality: 0%. Paraplegia: 0%. Branch occlusion in hospital: 0%. Median follow-up: 36.0 months (range 6–72). Endoleak during follow-up: 0.7% (1 case). Branch occlusion during follow-up: 0.7% (1 case). Deaths during follow-up: 2 cases. Complete false-lumen thrombosis: 99.3% (140 cases). Significant aortic remodeling at the aortic isthmus and pulmonary artery bifurcation (*p* < 0.01)
[16]	Cratos	Multicenter prospective	89	TBAD	Technical success: 100%. 30-day mortality: 1.1%. Stroke: 1.1%. Spinal cord ischemia: 1.1%. Median follow-up: 369 days. Deaths during follow-up: 2/89 (2.2%), both non-aortic-related. Reintervention: 1/89 (1.1%) for RTAD ^2^. Endoleak: 3 cases total; 2 resolved spontaneously, 1 associated with RTAD ^1^/type Ia endoleak. 12-month CTA available in 81 patients. Branch patency at 12 months: 95.1% (77/81). Complete thrombosis at the stent-graft-covered segment at 12 months: 92.6% (75/81)
[20]	Castor	Multicenter retrospective	180	TBAD	Technical success: 100%. 30-day/in-hospital mortality: 2.8% (5/180). Stroke: 1.7% (3/180), all within 30 days. Early reintervention: 0.6% (1/180). Median follow-up: 18 months (IQR 13–24). Deaths during follow-up: 2.8% (5/180). Late reintervention: 1.7% (3/180). Complete false-lumen thrombosis at 1 year in the stent-graft-covered segment: 97.1%.
[6]	TBE	Multicenter prospective	40	Aortic arch aneurysm	Mean follow-up: 1408 ± 552 days in zone 2 and 1187 ± 766 days in zone 0/1. Device migration/fracture/aortic rupture at 3 years: 0% in both arms. Freedom from reintervention in zone 2: 97% at 1 and 3 years. Side-branch occlusion in zone 2: 2 cases. Aneurysm enlargement >5 mm in zone 2: 2 cases, without documented endoleak or reintervention. Freedom from death in zone 2: 90% at 1 year; 84% at 3 years. Zone 0/1: 0 reinterventions, 0 branch-patency loss, 0 aneurysm enlargement at 3 years. Cerebrovascular events during follow-up: 3 cases in zone 0/1 (2 unrelated; 1 of unknown relationship). Deaths during follow-up in zone 0/1: 2, both unrelated to procedure or aneurysm.
[27]	Castor vs. standard TEVAR	Single-center retrospective	73	TBAD	ICU ^3^ stay: 44.02 ± 6.62 h vs. 47.26 ± 6.14 h (*p* = 0.03). Postoperative hospital stay: 12.53 ± 2.14 vs. 13.87 ± 2.23 days (*p* = 0.01). Total hospital stay: 20.47 ± 3.38 vs. 22.59 ± 3.28 days (*p* = 0.008). Total early complication rate: 10.53% vs. 31.43% (*p* = 0.03). In-hospital/30-day mortality: no significant difference between groups (*p* = 0.94). Secondary intervention: 2.63% vs. 25.71% (*p* = 0.01). Stent remaining in position: 100.0% vs. 80.0% (*p* = 0.01). New intimal tears at stent edge: 5.26% vs. 25.71% (*p* = 0.02). Improved without further deterioration: 97.37% vs. 80.00% (*p* = 0.046).
[17]	TBE	Single-center retrospective	55	Mixed	Preoperative spinal drain: 23/55 (41.8%). Technical success: 100%. Operative mortality: 3.6% (2/55). Stroke: 0%. Permanent paralysis: 0%. Paraparesis: 1/55 (1.8%), resolved after spinal drain placement. Renal failure requiring dialysis: 1/55 (1.8%). Median follow-up: 366 days (IQR 189–444). Endoleak during follow-up: 9/55 (16.4%). Reintervention for endoleak: 6/55 (10.9%)—5 endovascular, 1 open. Linearized all-cause mortality: 11.0% per patient-year
[18]	Cratos	Case report	1	Late TEVAR migration/ aneurysm	Index complication: 50 mm distal migration of the proximal TEVAR with type Ia endoleak in tortuous aortic anatomy. Treatment: proximal relining with Cratos and LSA preservation. Completion angiography: correct positioning and secure proximal sealing. 1-month CTA: branch patency preserved and no endoleak. Clinical relevance: first reported Western Cratos implantation and first reported use in aneurysmal disease
[33]	Castor + STABILISE ^4^	Case series	3	TBAD	Technical success: 100%. LSA preservation: 100%. Periprocedural complications: 0%. Reintervention: 0%. Complete aortic remodeling/false-lumen thrombosis reported
[25]	TBE	Single-center retrospective	40	Mixed	Proximal cuff used: 48%. Distal extension used: 83%. Prior ascending/arch repair: 60%. Planned as part of complete thoracoabdominal repair: 45%. Technical success: 100%. 30-day mortality: 2.5%. 30-day stroke: 5.0%. 30-day reintervention: 0%. 30-day endoleak: 7.5% (type II: 5.0%; unknown: 2.5%). Mean follow-up: 6.6 months. Side-branch patency at follow-up: 100%
[21]	Castor	Multicenter retrospective	106	Mixed	Technical success: 98.1% (104/106). Surgical success: 93.4% (99/106). Reintervention: 2.8% (3/106). Retrograde dissection: 1.9% (2/106). Type I endoleak: 1.9% (2/106). New dSINE ^4^: 2.8% (3/106). Branch patency: 100%. Mortality: 1.9% (2/106). Mean follow-up: 29.1 ± 17.7 months. 2-year cumulative survival: 91.0% ± 3.1%. 2-year cumulative branch patency: 96.2% ± 2.2%. 2-year freedom from stent-related reintervention: 93.2% ± 2.8%. No significant difference between zone 1 and zone 2 proximal landing in stent-related complications, branch patency, endoleak, reintervention, or mortality.
[50]	Castor	Single-center retrospective	10	Mixed	Technical success: 100%. Perioperative mortality: 0%. Stroke: 0%. Acute myocardial infarction: 0%. Renal failure: 0%. Left arm ischemia: 0%. Bird-beak: 0%. Type I endoleak: 0%. Type II endoleak: 0%. Type III endoleak: 0%. Secondary endovascular intervention during follow-up: 0%. Stent migration during follow-up: 0%. Mean follow-up: 1 year. Stent/branch patency maintained at follow-up
[11]	Castor	Multicenter retrospective	23	Mixed	Technical success: 96% (22/23). Median follow-up: 359 days (147–664). Side-branch patency: 95%. 30-day major adverse events: 0%. Perioperative stroke: 0%. Spinal cord ischemia: 0%. Perioperative mortality: 0%. Reintervention during follow-up: 2 cases, both for dSINE ^4^. Death during follow-up: 1 case, due to myocardial infarction at 92 days
[7]	TBE vs. TEVAR+ CSB ^5^	Single-center retrospective	125	Mixed	Proximal cuffs required: 14.7%. 30-day permanent cerebrovascular accident was highest in zone 0: 20%. 30-day myocardial ischemia was highest in zone 1: 20%. Overall survival at 1 year: 80.4%. Type Ia endoleak at 1 year: 1.3%. Sac regression at 1 year: 60%. False-lumen thrombosis at 1 year: 100%. Branch patency at 1 year: 100%. Zone 2 comparison: TBE *n* = 50 vs. TEVAR + LSAB *n* = 94. TBE was associated with lower postoperative AKI (OR 0.23; 95% CI 0.04–0.99; *p* = 0.048). TBE was associated with lower myocardial ischemia (OR 0.12; 95% CI 0.01–0.98). Length of stay was shorter with TBE (β −1.7 days; 95% CI −2.4 to −1.1; *p* < 0.001). Procedure time was shorter with TBE (β −54 min; 95% CI −71 to −38; *p* = 0.001). Contrast use was lower with TBE (β −42 mL; 95% CI −61 to −23; *p* < 0.001). Sac regression at 1 year was greater with TBE (OR 7.48; 95% CI 1.43–12.27; *p* = 0.02). Freedom from reintervention was greater with TBE (HR 0.26; 95% CI 0.6–0.92; *p* = 0.015).
[37]	TBE	Single-center retrospective	5	Acute aortic pathology	Technical success: 100%. Neurologic events: 0%. Cardiac events: 0%. Mean operative time: 122 min. 3-month CTA follow-up: satisfactory in all cases.
[22]	Castor	Multicenter retrospective	32	TBAD	Technical success: 96.88% (31/32). Mean operative time: 87.44 ± 10.89 min. Mean contrast volume: 125.31 ± 19.30 mL. Neurologic complications: 0%. Perioperative mortality: 0%. Mean hospital stay: 7.84 ± 3.20 days. Mean follow-up: 68.78 ± 11.26 months. Non-aortic deaths during follow-up: 4/32 (12.5%). LSA patency at follow-up: 100% (28/28 available imaging). Immediate type I endoleak: 1/32 (3.12%). Type II endoleak: 0%. RTAD ^2^: 0%. dSINE ^4^: 0%
[23]	Castor	Meta-analysis	415	TBAD	Pooled technical success: 97.5%. Intraoperative endoleak: 0.1%. Intraoperative LSA patency: 99.52%. Early type I endoleak: 1.6%. 30-day mortality: 0.96%. Early reintervention: 0.9%. Perioperative stroke: 0%. 1-year survival: 99.7%. LSA patency: 99.3% at 6 months, 97.58% at 1 year, 95.23% at 2 years. Endoleak during follow-up: 0.3%. Left upper extremity ischemia during follow-up: 0.5%. LSA stent deformation/stenosis during follow-up: 2.2%. Quality of evidence: low to very low.
[29]	Castor	Single-center retrospective	29	TBAD	Technical success: 96.6% (28/29). Mean operative time: 106.7 ± 38.4 min. Perioperative mortality: 0%. Stroke: 0%. Paraplegia: 0%. Access-route complications: 3.4% (1/29). Median follow-up: 3.0 months (IQR 1.5–10.1). Branch patency during follow-up: 100%. Endoleak during follow-up: 3.4% (1/29). RTDA ^2^: 3.4% (1/29). dSINE ^4^: 13.8% (4/29). Stent migration: 0%. Thoracic remodeling: stented thoracic true lumen 11.2 → 26.6 mm, false-lumen 23.8 → 7.9 mm, with complete false-lumen thrombosis in 89.7% at follow-up. Abdominal remodeling: 33.3% showed true lumen growth, whereas 66.7% had true lumen stabilization/shrinkage
[9]	Castor vs. chimney vs. fenestration	Single-center retrospective	133	Acute aortic pathology	Technical success: 98.0% (Castor), 97.62% (chimney), 95.12% (fenestration). In-hospital mortality: 0% (Castor), 4.8% (chimney), 4.9% (fenestration). Stroke: 2.0% (Castor), 0% (chimney), 0% (fenestration). Spinal cord ischemia: 0% in all groups. Immediate endoleak: 4.0% (Castor), 7.1% (chimney), 4.9% (fenestration). Bird-beak configuration: 56.0% (Castor), 23.8% (chimney), 29.3% (fenestration); *p* = 0.003. LSA patency during follow-up: 96.0% (Castor), 95.2% (chimney), 95.1% (fenestration). LSA occlusion during follow-up: 2 cases in each group, all asymptomatic. Aorta-related reintervention: 2 cases in each group. Aorta-related mortality: 1 case (Castor), 2 cases (chimney), 2 cases (fenestration); no significant difference. Complete false-lumen thrombosis in the stented thoracic segment at follow-up: 45.0% (Castor), 31.3% (chimney), 38.7% (fenestration). Aortic remodeling remained favorable in all three groups during the first 2 years, with no significant between-group difference.
[49]	Castor	Multicenter retrospective	21	Mixed	Overall survival: 95.24% (20/21). Technical success: 90.48% (19/21), defined as correct Castor implantation with patent LSA branch and no endoleak. Type I endoleak after the procedure: 9.52% (2/21). Bird-beak: 9.52% (2/21). Access-site hematoma: 4.76% (1/21). Pseudoaneurysm: 4.76% (1/21). Access iliac artery rupture: 4.76% (1/21). Mean follow-up: 14 months (range 1–40). Reintervention for type I endoleak during follow-up: 1/21 (4.76%). LSA branch thrombosis requiring reintervention: 1/21 (4.76%). Subsequent branched endovascular aortic repair for unfavorable visceral aortic remodeling/rapid aneurysmal degeneration: 2/21 (9.52%)
[47]	Cratos	Case series	2	TBAD	Technical success: 100%. Complete exclusion of the primary entry tear: 100%. LSA flow preserved in both cases. Endoleak during follow-up: 0%. Neurological complications: 0%.

^1^ Blunt thoracic aortic injury; ^2^ Retrograde type A dissection; ^3^ Intensive Care Unit; ^4^ Distal Stent graft–induced new entry; ^5^ Carotid-Subclavian bypass.

**Table 3:** Feasibility studies included in the review and their key findings.

Ref.	Device(s)	Population	N	Main Feasibility Finding
[26]	Castor vs. TBE	Acute TBAD	100	Castor suitability 82% vs. TBE 22% off-the-shelf; tapering is central
[32]	Castor	Prior zone 2 TEVAR cohort	72	Feasibility 68.1%; large LSA diameter major cause of exclusion
[31]	TBE	BTAI ^1^	66	Only ~56% met IFU criteria in trauma
[28]	TBE	Mixed pathology needing zone 2 seal	93	AF 92%, true feasibility 85%; iliac access and sex differences important

^1^ Blunt thoracic aortic injury.

**Table 4:** Main advantages and limitations of the different devices.

Domain	Castor/Cratos: Main Advantages	Castor/Cratos: Main Limitations	Gore TAG TBE: Main Advantages	Gore TAG TBE: Main Limitations
Evidence base	Broader and older literature, with the most mature dedicated evidence in TBAD, including prospective multicenter and long-term data.	Evidence is predominantly dissection-centered and Chinese; Western series remain smaller and shorter in follow-up.	Strong Western evidence base, including prospective midterm aneurysm data and growing real-world multicenter experience across zones 0–2.	Younger literature, less mature in acute TBAD, with many post-commercial reports still limited in follow-up.
Pathology fit	Particularly suited to TBAD, especially in short proximal landing zones and in anatomies with marked proximal-to-distal mismatch.	Less extensively studied in aneurysm-predominant Western arch anatomy; branch diameter ceiling may limit use in large LSAs.	Particularly attractive in distal arch aneurysm and mixed arch pathology, with strong branch durability and broad Western applicability; may also be advantageous in small native thoracic aortas, such as in trauma.	Lower off-the-shelf suitability in acute TBAD when strict anatomical criteria are applied, mainly because of distal tapering mismatch.
Aortic diameter treatment envelope	Proximal diameters 26–44 mm and distal diameters 20–44 mm, allowing tapered main-body configurations with up to 8 mm tapering.	Does not extend to very small proximal aortic diameters, which may limit use in selected young trauma patients with small native aortas.	IFU-based aortic sizing ranges from 16 mm to 42 mm, which may be advantageous in small, non-dilated thoracic aortas.	No true tapered branched main body; relevant proximal-to-distal mismatch may require adjunctive distal extensions, particularly in acute dissection.
Short zone 2 anatomy/proximal landing requirement	Major advantage in very short LCCA–LSA anatomies, because the branch can arise very close to the proximal graft edge; particularly valuable in dissection.	Requires meticulous planning and precise rotational alignment to fully exploit this short-offset advantage.	Performs well in appropriately sized, more regular zone 2 anatomies and is supported by formal IFU-driven planning criteria.	Requires a longer proximal zone 2 segment than Castor/Cratos; short LCCA–LSA distance is a frequent cause of non-feasibility.
LSA branch diameter range	Adequate for most dissection anatomies and standard LSA calibers.	Branch diameter is generally limited to 14 mm; larger LSAs may require adjunctive covered stenting or may be anatomically unsuitable.	Clear advantage in large LSAs, with branch options up to 20 mm and treated-vessel ranges up to 18 mm in the IFU.	Greater branch flexibility is achieved through a modular side-branch system rather than an integrated construct.
Core architecture	Integrated unibody design with antegrade branch; behaves as a single construct and avoids an arch-level modular junction.	Castor may be more technically demanding to orient and deploy in tortuous arches.	Modular design provides versatility and broad applicability across the contemporary arch spectrum.	Introduces at least one component junction and requires a separate bridging side branch into the LSA.
Sealing and fixation concept	The integrated antegrade branch may contribute to proximal construct stabilization and reduce dependence on a long proximal neck.	Despite this conceptual advantage, proximal seal still depends on anatomy and deployment accuracy; bird-beak and type Ia endoleak remain possible.	Excellent branch patency and reproducibility in aneurysm and mixed arch cohorts.	The retrograde branch does not contribute to proximal sealing in the same way; proximal landing requirements therefore remain more demanding.
Modularity/number of components	Single integrated arch-level construct, without the need for a separate bridging branch component.	Less versatile than a modular arch platform when more proximal arch extension is required.	Major advantage in versatility: usable across zones 0–2 and compatible with adjunctive components when needed.	Modularity increases procedural steps and introduces junction- and overlap-related failure modes; distal extensions are often required in real-world practice.
Maneuverability/bird-beak control	Cratos improves maneuverability and delivery-system control versus Castor and was designed to improve proximal conformability and reduce bird-beak formation.	Castor may be less forgiving during rotational alignment and arch navigation, especially in tortuous anatomy.	Familiar modular Gore platform for many arch operators and reproducible in experienced centers.	No dedicated bird-beak-control feature analogous to that proposed for second-generation Cratos has been described in the current TBE literature.
Deployment control	Integrated design may reduce arch-level component interaction during release; Cratos further improves deployment control versus Castor.	Castor still requires careful rotational orientation and precise branch alignment.	Standardized modular deployment strategy with a well-described procedural workflow.	Accurate release requires meticulous stabilization; forward or backward movement during deployment may lead to inaccurate positioning.
Femoral access profile	Castor has a stable 24F profile, and Cratos reduces this to 22F up to 34 mm, which may be advantageous in borderline iliofemoral anatomy	Large-profile femoral access is still required, especially for Castor, and may remain limiting in small or diseased iliac axes.	Smaller TBE configurations may have favorable access profiles.	Large-diameter TBE configurations may require up to 26F introducers, reducing feasibility in women and hostile iliac anatomy.
Upper-extremity access	Familiar and effective branch access strategy, widely used in published Castor series.	Upper-extremity access is typically larger (around 7–8F), which may more often prompt limited surgical brachial exposure.	Usually requires smaller upper-extremity working access (4–5F), facilitating percutaneous radial or brachial strategies.	-
Regulatory/ logistic profile	In China, Castor has effectively functioned as an off-the-shelf platform since approval by CFDA; (NMPA). In selected European markets, some common configurations may be available with relatively short lead times.	Despite this practical availability in selected settings, Castor is not approved as an off-the-shelf platform in Europe. Current European documentation still describes it as a custom-made device supplied on a named-patient basis only Cratos has so far been reported clinically under a custom-made framework rather than a standard off-the-shelf CE pathway. Not available for sale in the United States.	TBE is a true off-the-shelf modular platform, supported by FDA approval and CE marking.	-

## Data Availability

No new datasets were generated or analyzed in this study. All data discussed in this review are available in the cited published articles.

## Supplementary Materials

The following supporting information can be downloaded at: https://zenodo.org/records/19003812?token=eyJhbGciOiJIUzUxMiJ9.eyJpZCI6IjIyYWE2YThjLWQ1ZjYtNGM1OS1iNTBiLWZkNTcxMjU2N2VjYSIsImRhdGEiOnt9LCJyYW5kb20iOiI4YzgwNDRhZjM1OTAwNWQ1NGMyYTFhOTFlZGJiNGI1NCJ9.Htcqhn-SQ4lhP0vxbQ20MIx012hv1Qg9J_sJTFspKHMlgOkRf1LxScASyw8aWnynKQbwU5cnzhs_VTNjL_sbag (accessed on 29 March 2026); Video S1: Castor Deployment video; Video S2: Cratos Deployment video; Video S3: TBE Deployment video.

## Abbreviations

The following abbreviations are used in this manuscript:

3D	Three-dimensional
AF	Anatomical Feasibility
AKI	Acute Kidney Injury
BEVAR	Branched Endovascular Aortic Repair
BTAI	Blunt Thoracic Aortic Injury
CE	European Conformity
CFDA	China Food and Drug Administration
CSB	Carotid-Subclavian Bypass
CT	Computed Tomography
CTA EVAR	Computed Tomography Angiography Endovascular Aneurysm Repair
FDA	Food and Drug Administration
IF	Iliac Feasibility
IFU	Instruction For Use
IMH	Intramural Hematoma
LCCA	Left Common Carotid Artery
LSA	Left Subclavian Artery
LVA	Left Vertebral Artery
MAE	Major Adverse Event
MDR	Medical Device Regulation
MI	Myocardial Infarction
NMPA	National Medical Products Administration
PAU	Penetrating Aortic Ulcer
PRISMA	Preferred Reporting Items for Systematic Reviews and Meta-Analyses
RTAD	Retrograde Type A Dissection
SCI	Spinal Cord Ischemia
SINE	Stent Graft-Induced New Entry
STABILISE	Stent-Assisted Balloon-Induced Intimal Disruption and Relamination in Aortic Dissection Repair
TBE	Thoracic Branch Endoprosthesis
TBAD	Type B Aortic dissection
TEVAR	Thoracic Endovascular Aortic Repair
TF	True Feasibility
